# Fully inkjet-printed microfluidics: a solution to low-cost rapid three-dimensional microfluidics fabrication with numerous electrical and sensing applications

**DOI:** 10.1038/srep35111

**Published:** 2016-10-07

**Authors:** Wenjing Su, Benjamin S. Cook, Yunnan Fang, Manos M. Tentzeris

**Affiliations:** 1 Georgia Institute of Technology, School of Electrical and Computer Engineering, Atlanta, GA 30332-250, USA; 2 Texas Instruments, Kilby Labs, Dallas, TX 75243, USA; 3 Georgia Institute of Technology, School of Materials Science and Engineering, Atlanta, GA 30332-245, USA

## Abstract

As the needs for low-cost rapidly-produced microfluidics are growing with the trend of Lab-on-a-Chip and distributed healthcare, the fully inkjet-printing of microfluidics can be a solution to it with numerous potential electrical and sensing applications. Inkjet-printing is an additive manufacturing technique featuring no material waste and a low equipment cost. Moreover, similar to other additive manufacturing techniques, inkjet-printing is easy to learn and has a high fabrication speed, while it offers generally a great planar resolution down to below 20 *µ*m and enables flexible designs due to its inherent thin film deposition capabilities. Due to the thin film feature, the printed objects also usually obtain a high vertical resolution (such as 4.6 *µ*m). This paper introduces a low-cost rapid three-dimensional fabrication process of microfluidics, that relies entirely on an inkjet-printer based single platform and can be implemented directly on top of virtually any substrates.

Due to its capability of manipulating extremely small quantities of liquids, microfluidics technology has been widely used in biomedical sensing, chemical assay, manufacturing control and other lab-on-chip applications in the past decades^1^,^2^. Novel fabrication methods of microfluidics have been researched and reported in academia as well as in industry, and every emergence of a new lower cost fabrication technique usually became the impetus to even further blooming microfluidics.

In the early 1990s, microfluidics were firstly fabricated in silicon and glass using photolithography^3^, a conventional planar manufacturing technique which patterns features by using light-sensitive materials (photoresist) and masks. Photolithography features an unbeatable high resolution but requires costly experiments and cleanroom environment, thus it is too expensive for numerous applications. At the end of the last century, Soft-lithography technique was invented by pouring a thermosetting material, Polydimethylsiloxane (PDMS), into a replica mold and casting the microfluidics^4^,^5^. Soft-lithography has a much lower cost and a more rapid production speed compared to old photolithography method. Moreover, it facilitates designs of high flexibility and high optical transmittance, thus having established soft-lithography as the most common microfluidics fabrication process. However, this mold-based method usually needs photolithography to fabricate the mold. Thus, soft-lithography is a semi-cleanroom process which still heavily relies on costly photolithography techniques. Furthermore, the production quantity needed for each specific microfluidics design is normally very small, even as low as one. In this situation, the mold-based approach is very costly and may not be the best option^6^. On the other side, the needs of rapid production of low-cost and disposable microfluidic devices have been dramatically growing with the development of distributed/point-of-care healthcare, complex laboratory tests, and other lab-on-chip applications. Recent years have witnessed an increasing number of novel and low-cost fabrication approaches being proposed to address these issues.

Additive manufacturing (AM) has been a novel fabrication approach that has attracted a significant attention in recent years as it can rapidly fabricate structures at a low cost with no waste. People have already tried to utilize numerous three dimension (3D) printing techniques, the most well-known AM subclass, in microfluidics^6^,^7^,^8^,^9^,^10^,^11^,^12^,^13^,^14^,^15^. Most 3D printing-based approaches only print the mold for casting (e.g. soft-lithography)^7^,^8^, which partially solves the problem of soft lithography. Other moldless approaches directly print the microfluidics with various 3D printing techniques, which effectively enable the rapid prototyping of microfluidics^10^,^11^,^12^,^13^,^14^. However, currently most 3D printers feature generally low resolutions and large minimal feature size^16^, which results in a typical large cross-section area of 3D printed microfluidcs channels at least on the level of 5 × 10^4^ *μm*^2^ 8,9,10,11,12,13.

Another AM technique, inkjet-printing, has been recently involved in microfluidics fabrication including paper microfluidics^17^,^18^,^19^,^20^, inkjet-printing bonding microfluidics^21^,^22^,^23^,^24^ and many other approaches^25^,^26^,^27^,^28^. Inkjet-printing is a drop-on-demand (DOD) liquid phase material deposition technique and is a highly commercialized technique utilized in most office printers^29^. It usually involves the ejection of a fixed quantity of ink from the nozzle in pulses and results in drops fired onto the substrate. By using inkjet printing, the cost of time and resources for the microfluidics fabrication can be decreased significantly. Figure 1 shows a comparison of costs and production volumes of different microfluidics fabrication methods^17^. Compared to 3D printing, inkjet-printing normally costs less for the same planar resolution and allows for a much smaller printable layer thickness leading to a better vertical resolution.

Paper microfluidics, or paper-based analytical devices (*μ*PADs), have attracted plenty of attention recently. By inkjet-printing hydrophobic boundaries (usually with wax^18^,^19^ or polymer^17^,^20^) of channels on hydrophilic paper, plenty of paper microfluidics have been prototyped, which fit the needs of various chemical and biomedical assays. However, most paper microfluidics obtain virtually only 2D structures and can only be integrated vertically by stacking papers and tapes (or other adhesives)^20^, which has been reported to suffer from numerous drawbacks, including errors in alignment and interlayer interference due to the paper fibers. On the one side, paper microfluidics have the inherent advantage that the paper fibers in the channel can hold the reagents within themselves. On the other side, as channels cannot be fabricated to be completely empty and completely sealed in four directions, the application of paper microfluidics has been limited up to now.

Besides paper microfluidics which print only the barriers, inkjet-printing has been also used in various steps in microfluidics fabrication process including sealing the channel^21^,^22^,^23^,^24^; building the mold for PDMS replication^25^,^26^; patterning the mask for channel etching^27^; printing the sidewall between two substrates^28^. It’s easy to notice that these approaches still require co-fabrication with other fabrication techniques, such as laser etching^21^,^22^,^23^, 3D printing^24^, wet etching^27^ or soft-lithography^25^,^26^, which complicates the fabrication process flow and may increase the cost significantly. In this paper, we propose, characterize and implement fully inkjet-printed microfluidics for the first time. This process relies only on inkjet-printing technology and enables rapid fabrication of complex microfluidics networks with 3D configurations on virtually any substrate at an extremely low cost. Moreover, as inkjet-printing conductive materials have been extensively used in low-cost flexible electronics^30^,^31^,^32^, the integration of microfluidics and electronics becomes very convenient using the proposed inkjet-printing, and fully inkjet-printed electronic sensors are prototyped and presented in this paper for proof-of-concept demonstration and verification purposes.

## Results

### Fabrication process

In the proposed approach, 3D microfluidics devices are fabricated by inkjet-printing different materials level-by-level. Two different polymer inks are involved: the SU-8 ink, which constructs the microfluidic channel, and the Poly(methyl methacrylate) (PMMA), which supports the structure during curing and is washed out afterwards. Due to its highly chemical resistance, SU-8 can remain almost intact during etching and after interaction with most organic and inorganic solvents under test inside the channel. Poly(methyl methacrylate) (PMMA), on the other side, is easy to wash out with etching chemical solvents. Due to the fact that PMMA ink is always printed on SU-8, no matter what substrate is used and how many levels the structure has, a great consistency and an excellent control of the channel shape can be achieved. Without loss of generality and as a proof-of-concept demonstration, we used the Dimatix DMP-2831 printer^33^, a cost-effective and easy-to-use typical industrial inkjet-printer with 5 *μ*m minimal pixel size/resolution and a cost under $30,000. The maximum build size of this printer is 210 mm × 315 mm × 50 mm, which should be big enough to accommodate any microfluidics chip and in most cases enabling the simultaneous fabrication of multiple chips.

**Figure 2:**
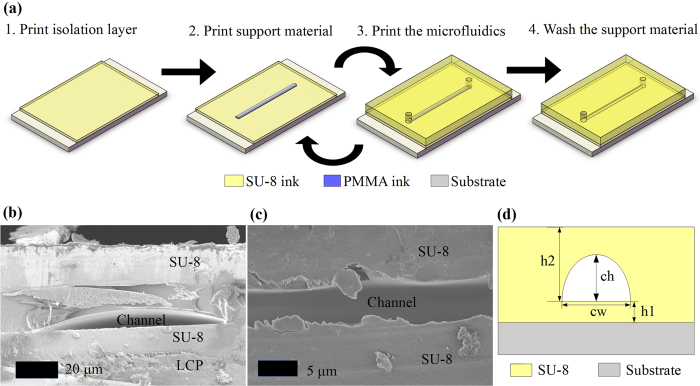
Fabrication process steps and cross-section views of inkjet-printed prototypes. (**a**) Process flow of the fully inkjet-printed microfluidics fabrication. (**b**,**c**) Cross-sectional SEM images of proof-of-concept microfluidic channel prototypes with a low (**b**) and a high (**c**) aspect ratios under different magnifications. (**d**) A sketch of a fabricated channel in a cross-section view along with dimension notations.

The printing process can be represented by the flow of the four steps in Fig. 2(a). Firstly, a thin level of SU-8 ink was printed on a substrate to isolate the substrate from the fluids in the microfluidic channel. For applications such as electrical sensing, this isolation can eliminate the unwanted contact of metals/conductors (if any) with the fluids under test. For porous substrates such as papers and corks, this isolation printing can fill small substrate holes/voids and provide a water-proof channel. For substrates with slightly uneven surfaces, this printing approach can even out the undesired roughness. This isolation printing is a preparatory step in the cases the substrate is not optimal, and thus it could be bypassed otherwise. Because of this step and the additive nature of inkjet-printing, this process is applicable to build 3D microfluidic structures on virtually any substrates or devices including glass, paper, silicon wafers, metal, plastic, and other existing microfluidics chips as well as packaging structures. Secondly, a patterned PMMA trace was deposited as a placeholder for the microfluidic channel topology. This printed pattern was the same as the designated microfluidics channel pattern, which would support all the materials deposited on top of the channel in the following steps. Thirdly, a thick SU-8 layer was printed which would constitute the microfluidic channel walls in the end of the process. The openings (inlet and outlet) of the microfluidic channel were preserved by the CAD pattern file. As the SU-8 ink was in liquid phase, it would even out the surface including the printed PMMA traces if enough amount was deposited, thus resulting in a structure with a flat surface. In the case of printing 3D structures, we simply repeated the second and third steps until the necessary numbers of levels were printed. Finally, after all levels were printed, the removal of the PMMA support material was carried out by an anisole solution bath, although several other alternative organic solvents could work as well. As etching relied on the diffusion of the etching solvent, we found that the etching time, which strongly depended on how narrow and long (e.g., cross-section area/length ratio) the channel was, can be significantly decreased by applying an ultrasonic bath. After the removal of the support material in the channel, a hollow channel embedded within the SU-8 polymer can be observed with the same shape as the printed PMMA pattern and scanning electron microscope (SEM) photos of the cross-section of proof-of-concept channel prototypes are shown in Fig. 2(b,c). The debris particles shown in the SEM images were probably SU-8 polymer. The cured SU-8 polymer was very brittle and easily broken during the cutting process for the demonstration of the cross section of the inkjet-printed microfluidic channel.

#### Inkjet-printing PMMA

**Figure 3:**
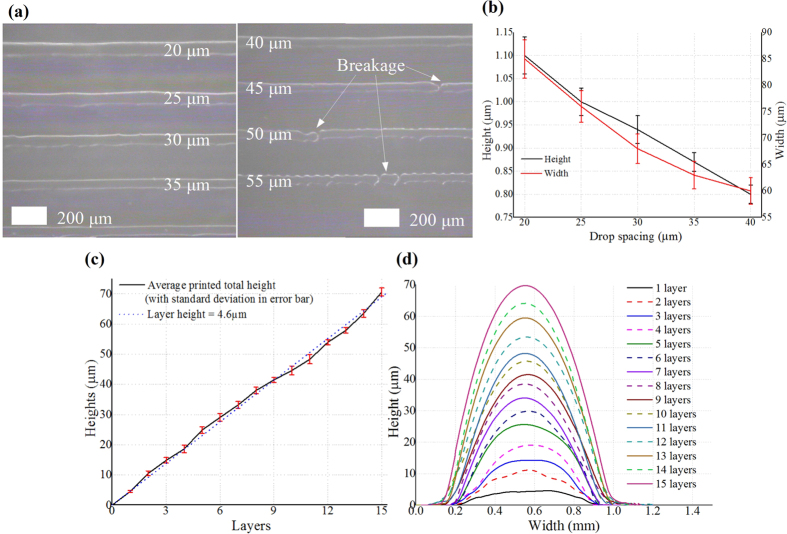
Performance of inkjet-printed PMMA. (**a**) Photos of “single-drop” (narrowest realizable width) printed PMMA lines for different drop spacing values. The line prototypes are 5 mm long and only a subsection is shown in this photo. (**b**) Meaasured average height and width with error bars of the printed PMMA line prototypes in (**a**). (**c**) Average height and error bar of separately printed 7 mm × 0.6 mm lines for 1 to 15 printed layers with individual layer thickness of 4.6 *μ*m. (**d**) Cross-sectional profile of the printed PMMA lines in (**c**) for different numbers of printed layers measured by a contact profilometer.

Inkjet-printing PMMA is the key step in this process, as it defines the horizontal (2D) pattern as well as the height of the microfluidic channel. A CAD file is used to define the 2D pattern which is the same as the top view of the desired pattern for each level. So every parameter of the channel except from the channel height, such as the channel width (*cw* in Fig. 2(d)), length and orientation, can be directly incorporated in the CAD file. The 2D patterns for each layer are then decomposed into a grid of 5 *μ*m × 5 *μ*m pixels by the printer and realized by a drop matrix with a fixed drop spacing, which defines the minimal resolution of the pattern. This drop spacing can feature any size larger than 5 *μ*m to successfully generate the drop matrix, but if it’s too small, inks may spread out due to too much material deposited per area, whereas if it’s too large, the pattern is printed at a low resolution and may introduce unwanted discontinuities as shown in Fig. 3(a). 20 *μ*m was found experimentally to be the optimal drop spacing value, effectively offering a 20 *μ*m resolution.

Nevertheless, a larger (>20 *μ*m) drop spacing means less deposited material per unit area thus allowing the fabrication of thinner lines and small features. To further investigate this fact, various single-drop line (consecutive single drops in one direction/line) prototypes were printed with different drop spacings, as shown in Fig. 3(a), featuring the realized height and width values shown in Fig. 3(b). For drop spacings larger than 40 *μ*m, line breakages happen thus spacings larger than this value should be avoided. Therefore, the smallest channel achieved by this process so far has been 60 *μ*m wide (“*cw*” in Fig. 2(d)) and 0.8 *μ*m high (“*ch*” in Fig. 2(d)) with 40 *μ*m drop spacing, which is the most miniaturized reported cross-section of a microfluidic channel fabricated with non-photolithographic AM methods to the best of our knowledge.

As the CAD pattern defines the horizontal channel pattern, the number of printed layers plays a dominant role in the control of the channel height (“*ch*” in Fig. 2(d)). In multilayer inkjet-printing, it is possible to accurately control the amount of the deposited material by simply printing the same pattern at the same location for multiple times, each one of which defines one additional layer. Consequently, we can realize various channel height values as the multiples of the individual printed layer thickness as shown in Fig. 3(c). In order to calibrate and verify the accurate control of the individual layer height, numerous 7 mm × 0.6 mm lines are printed for 1 to 15 layers respectively with measured results shown in Fig. 3(c,d). The layer height is reliably around 4.6 *μ*m with less than 0.4 *μ*m variation, which shows the excellent capability of patterning the channel with minor errors.

Due to the liquid nature of the ink, the cross-section of the printed trace features a semi-elliptical shape as shown in Figs 2(b–d) and 3(d). Similar to all other inkjet-printing processes, the cross-section shape of the printed pattern depends on both the ink curing conditions and the contact angle of the ink on the SU-8 substrate. The ink composition, the surface properties of the substrate (e.g. hydrophobic or hydrophilic) and the printing platform temperature mainly decide the contact angle. The lower limit of the fabricated aspect ratio is 10 for the printing setting discussed previously in this paper and is mostly controlled by the contact angle. Higher contact angle values lead to an increased curvature of the outline of of the cross-section and to a decreased minimum realizable aspect ratio. However, structures with aspect ratios lower than 10 could still be constructed performing several consecutive printing-curing processes.

#### Inkjet-printing SU-8

Similar to PMMA, a CAD file is used to define the 2D pattern of SU-8 prints, which in most case is simply a sheet with “through” holes functioning as the inlet/outlet of the microfluidics channel. The goal of this step is printing a thick and strong SU-8 layer to cover the PMMA traces and to support the channel structure in order to prevent any crack formation. Therefore, the excellent control of the SU-8 thickness, especially the thickness on top of the PMMA line, and the full cross-linking of the SU-8 are the keys for a successful microfluidic structure.

**Figure 4:**
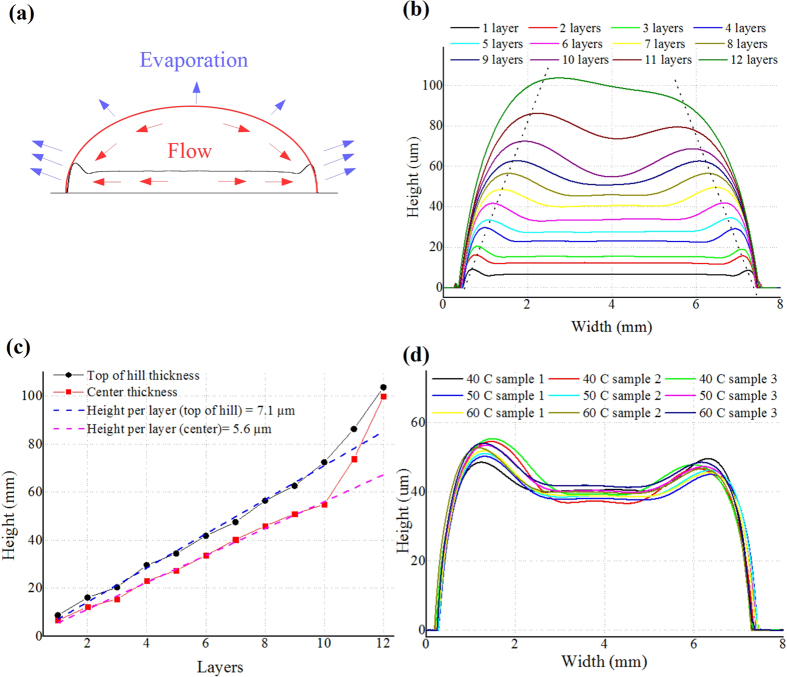
Features of inkjet-printed SU-8. (**a**) A sketch to demonstrate the “coffee ring” effect during ink curing in a cross-section view. The red curve stands for the deposited liquid-phase ink and the black line stands for the final shape after the solidification of the ink. The blue arrow shows the evaporation of the solvent and the red arrow shows the flows within the liquid-state ink during curing. (**b**) The profile of the inkjet-printed rectangular “pad” (7 mm × 7 mm) prototypes for a different numbers of layers. The two dotted lines mark the “coffee ring” hill-top trend for an increasing thickness. (**c**) Printed SU-8 thickness in the center of the “pad” (“center”) and at the hill-top of the “coffee ring” (“top of hill”) for different numbers of printed layers. (**d**) The profile of the inkjet-printed rectangular “pad” (7 mm × 7 mm for 7 layers) prototypes at various printing platform temperatures (40 °C, 50 °C and 60 °C; 3 samples at each temperature).

Numerous 7 mm × 7 mm rectangular “pad” prototypes (printed structures with much larger horizontal dimensions compared to height/thickness) for 1 to 12 layers were printed to characterize the SU-8 thickness for a different number of deposited layers. Unlike printing PMMA line, which was deposited along a narrow line, the required SU-8 usually occupied a much larger area, thus the “coffee ring” effect took place. This effect is named after the characteristic ring-like deposit along the perimeter of a spill of coffee and is a phenomenon frequently happening in the inkjet-printing; in the presented case coffee is replaced by the SU-8 ink. When drying a printed ink pattern, as shown in Fig. 4(a), the speed of evaporation is much higher at the edges of the pattern than at the center due to the higher surface area-to-volume ratio. This leads to flows from the center to the edges resulting in a basin-like topology^29^. As can be seen in the dashed line in the Fig. 4(b), there is an almost linear relation between the “hill” height and the width between the “top of hill” to edge, which is dominated by both the drying speed and the contact angle of the SU-8 ink on the underlying substrate. For the printing configurations utilized in this paper, this ratio is 55 *μ*m (height): 1 mm (width) at the edges of the printed “pad”. Figure 4(c) shows the fabricated thickness/height of different SU-8 layers for the 7 mm × 7 mm rectangular “pad” prototypes. The “coffee ring” effect could be easily observed as the “top of hill” thickness is higher than the center thickness. From 1 to 10 printed layers, the height per layer is reliably around 5.6 *μ*m per layer in the center and 7.1 *μ*m at the “top of hill” as shown in the dashed line in Fig. 4(c). When more layers are printed, the resulting solidified SU-8 performs closer to a “line” or “dot” (horizontal dimensions comparable or smaller than the height/thickness), which features a minimal “coffee ring” effect as Fig. 4(c) shows that the measured height in the center and at the “top of hill” are getting closer in value for an increasing number of printed layers (e.g. 11–12 layers) resulting in a semi-elliptical cross-section outline in Fig. 4(b) similar to the inkjet-printed PMMA discussed in previous section. Therefore, the height increment for each additional printed layer effectively stops following the dashed line in Fig. 4(c) in the situation of more than 10 layers printed. As most microfluidics devices are large and thin preferably with a flat top over the channels, “pad”-like printing with a sacrificing “coffee ring” or “hill” area is desired, which means the horizontal dimensions of the pattern to print (which may contain multiple channels) should be sufficiently larger than 36 times (=2 × (1 mm/55 *μ*m)) of the desired height.

The influence of printing platform temperature was investigated as shown in Fig. 4(d). Theoretically, the substrate temperature would change the contact angle of the ink and the coffee ring effect, while may lead to different shapes after drying. However, as the temperature of the printing platform is relatively low compared to the temperature of the hot plate used in curing process and printing time is short in most cases, the evaporation during printing is much smaller compared to the evaporation during the curing process. Therefore, the influence of the temperature of the printing platform is negligible. Numerous independent samples of 7-layers 7-mm-by-7-mm squares were printed at 40 °C, 50 °C and 60 °C, respectively, and their profiles were measured by a profilometer with results shown in Fig. 4(d) for three samples per temperature. There is no observed correlation between the temperature and the outcome shapes of the printed prototypes.

#### Inkjet-printing conductive materials

**Figure 5:**
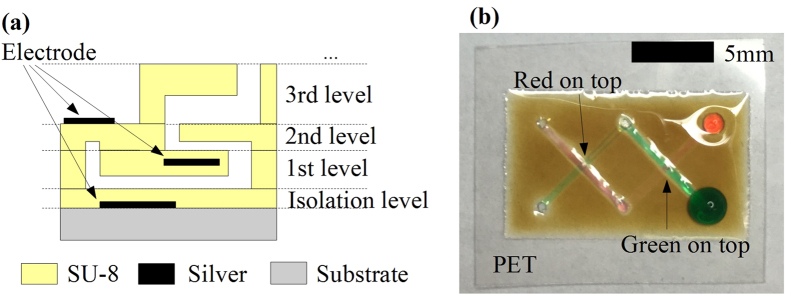
Sketch and prototype of a fully inkjet-printed 3D microfluidics structure. (**a**) Sketch of the side view of an arbitrary fully inkjet-printed 3D microfluidics structure with integrated conductors. (**b**) A photo of two fully inkjet-printed twisted 2-level 3D microfluidic channels. Due to the capillary effect, one channel is filled with red dyed water and the other is filled with green dyed water. The two channels are twisted with respect to each other: The green one is on top of the red one in the right half and is beneath the red one in the left half.

One of the main advantages of this process is that the fully inkjet printed microfluidics can be easily integrated with electronics through the inkjet-printing of conductive inks^22^,^30^,^32^,^34^ contrary to other microfluidics processes, such as soft-lithography or 3D printing, which normally cannot handle conductors. Effectively, by simply adding the extra step of inkjet-printing conductive inks, arbitrary conductive patterns can be embedded almost anywhere in 3D microfluidics structures as shown in Fig. 5(a). The conductive ink could be a nanoparticle-based ink, such as the silver nanoparticle ink presented in this paper, which features a superior conductivity (e.g. below 0.01 Ohm/square resistance) but needs sintering (e.g. at 150 °C), or reactive inks that can achieve good conductivity with no high-temperature sintering^34^. Every component in the microfluidics passive electronic sensors can be inkjet-printed on the same (single) platform enabling for the first time a plethora of applications in healthcare and bio assay.

#### 3D microfluidics

The proposed fabrication process can be used to fabricate fully 3D microfluidics structures similar to the one shown in Fig. 5(a) with a level-by-level mechanism similar to 3D printing. Every channel that is on top of another channel is defined as a new level as it needs to be printed after the completion of the bottom level. Each level is very independent in terms of printing, which means not only there are no limitations on what pattern is printed but also each level can be fabricated with a different level height/thickness (such as the 1st, 2nd and 3rd levels in Fig. 5(a) that feature three different level heights) without any extra effort. For the printing of each level, the fabrication process is the same as described above, except that instead of printing on the isolation level, the pattern is printed directly on the underlying layer, thus leading to 3D microfluidics configurations constructed of numerous single-level ones. In this way, the whole microfluidics structure is still constructed using the same material, SU-8. This feature differentiates the proposed inkjet-printing method from the other level-by-level approaches, such as stacking by inserting adhesive layers. Compared with 3D printer-based approaches, a similar functionality is achieved for a much better vertical resolution and a resulting much smaller realizable printable layer thickness. An example of of two fully inkjet-printed twisted 2-level microfluidic channel prototypes can be found in Fig. 5(b), that clearly demonstrates the excellent capability for the fabrication of complex 3D microfluidics topologies.

### Applications

The fabrication process presented above can be used in various microfluidics applications including but not limited to wearable sensors, biomedical assays, chemistry analysis and micro-fabrications as it is applicable to any structures ranging from simple single level channels to complex multilayer topologies. In this section, we present various fully inkjet-printed prototypes of both basic microfluidics structures and microfluidics sensors with integrated electronics.

#### Microfluidics structures

**Figure 6:**
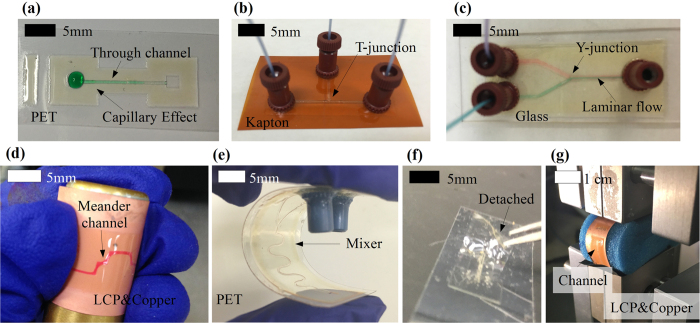
Examples of fully inkjet-printed microfluidics structures. (**a**) A straight microchannel on PET substrate. A drop of green-dyed water was dripped on one of the two channel openings and the capillary effect drove the water to enter the channel. (**b**) A T-junction and three microfluidic channels along with three installed connectors on Kapton substrate. (**c**) A Y-junction and three microfluidic channels along with three installed connectors on glass. One of the two inlets was fed with green-dyed water and another inlet was fed with red-dyed water. A laminar flow appeared when the two different color-dyed flows merged. (**d**) A meander microchannel on LCP substrate (coated by copper) bent in tension around a 14-mm-diameter rod with red-dyed water filling the channel. (**e**) A fully inkjet-printed microfluidics mixer on PET substrate bent in compression between two fingers. (**f**) A microfluidics device was detached from the substrate and was bent by a tweezer for a radius down to 0.5 mm. (**g**) A microfluidics device was bent by a TestResources four point bend tester for a radius of 1 cm.

Basic and flexible microfluidics structures, such as a straight channel (Fig. 6(a)), a T-junction(Fig. 6(b)), a Y-junction(Fig. 6(c)), a meander line(Fig. 6(d)) and a mixer(Fig. 6(e)) can be easily fabricated. Microfluidics devices fabricated with the proposed approach feature a very promising pressure handling and endurance. Water has been continuously pumped through the fabricated channels at 0.1 mL/min for over 10 hours, which shows a long-term water exposure capability. The printed microfluidic channels can operate at relatively high throughputs, such as up to 2 mL/min for a 0.03 mm^2^ cross-section area channel, which demonstrates a good pressure handling capability. As the sizes (cross-section areas) of the fabricated channels are typically in the range of 38 to 6 × 10^4^ *μ*m^2^, many microfluidics physical phenomena, such as the capillary effect and the laminar flow, can be observed with fabricated devices such as shown in Figs 5(b) and 6(a,c).

Five different substrates, namely PET (poly(ethylene terephthalate)) (Figs 5(b) and 6(a,e)), Kapton (polyimide film) (Fig. 6(b)), Glass (Fig. 6(c)), LCP (Liquid Crystal Polymer) (Fig. 2(b,c)) and copper cladded LCP substrate (Fig. 6(d,g)), are utilized respectively, demonstrating that this process can be applied to virtually every substrate. Among them, PET, Kapton and LCP are flexible polymer sheets and the microfluidics devices fabricated on them exhibit a very good flexibility in both bending directions as shown in Fig. 6(d,e). As a proof-of-concept demonstration, a microfluidics channel printed on 184 *μ*m-thick copper cladded LCP substrate was tested with a TestResources four point bend tester as shown in Fig. 6(g). Minimal bending radii achieved without damage are 7 mm in tension and 6 mm in compression. The microfluidics device was bent to a radius of 1 cm for 1000 times in tension and 1000 times in compression (2000 times in total) without any damage. It has to be stressed that the process is not limited to the substrate while the flexibility largely depends on the substrate flexibility and thickness. For the same microfluidics structure, a better bending capability may be achieved by using a more flexible and thinner substrate. When detached from the substrate, the printed microfluidics devices can be bent down to at least 0.5 mm, as shown in Fig. 6(f). However, as the detached microfluidics devices are super thin, they could be hard to handle and very fragile. Overall, the fully inkjet-printed microfluidics show an excellent flexibility for applications such as wearable sensors.

The microfluidics devices are constructed using a single material, SU-8, which is an excellent material for microfluidics^35^. SU-8 features an outstanding chemical resistance and biocompatibility^36^ which enables numerous chemical and biomedical applications. The high optical transmittance of SU-8 also enables transparent microfluidics devices for easier channel observation and optical applications^37^. The transparency of the SU-8 thin film is above 90% at visible light range^38^. Caution need to be paid in the exposure amount in the step of crosslinking the SU-8 as over-exposed SU-8 usually features a brown color as shown in Fig. 5(b), which may affect its transparency. Due to the hydrophobic property of the SU-8, the microfluidic performance of fluid (water-based) is very similar to the PDMS channels.

#### Electronic integrated applications

As mentioned above, inkjet-printing technique is widely used in fabricating low-cost flexible electronics in frequency ranges up to the radio frequency (RF) and millimeter-wave (mmW); thus, it would be straightforward to integrate inkjet-printed electronics with microfluidics fabricated with the proposed method, which makes microfluidics sensors with integrated electronics an excellent application of this fabrication process. Various examples of fully inkjet-printed microfluidics-based tunable electronics and sensors with integrated electronics are presented in this section.

**Figure 7:**
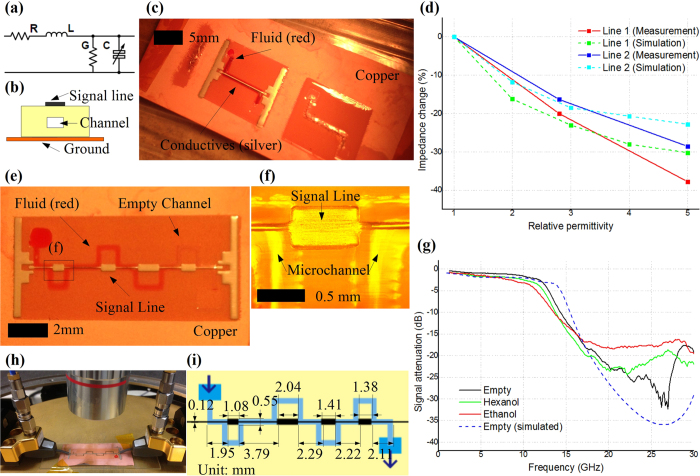
Fully inkjet-printed microfluidics sensors. (**a**) The equivalent circuit of the proposed fully inkjet-printed liquid-reconfigurable microstrip line. The variable capacitance stems from the varying permittivity of the liquid inside the microfluidics channel. (**b**) A sketch of the cross-section view of the liquid-reconfigurable microstrip line. (**c**) A photo of the proposed fully inkjet-printed liquid-reconfigurable microstrip line prototype. (**d**) Impedance vs. “liquid” permittivity relationship (simulated and measured) for the two fully inkjet-printed liquid-reconfigurable microstrip line prototypes in Table 1, effectively defining the sensor sensitivity. (**e**) A photo of the fully inkjet-printed low-pass filter-based microfluidics sensor prototype. (**f)** Enlarged under-telescope view of the zone within the rectangle in (**e**). (**g**) Measured signal attenuation when different fluids are fed to the channel as well as for an empty channel along with the simulated attenuation for an empty channel. (**h**) A photo of measuring the sensor with a probe station. A red-dyed water drop was deposited to the inlet of the channel to fill the channel through the capillary effect. Two ground-signal-ground (GSG) probes were in contact with the sensor prototype and were connected to a VNA via SubMiniature version A (SMA) cables. (**i**) A schematic of the topology of the low-pass filter-based microfluidics sensor along with dimensions.

In nature, fluids feature a wide permittivity (*ε*_*r*_) distribution at microwave frequencies and a lot of potential information about the fluids, such as the concentration and the mixing ratio, can extracted sensing the permittivity changes^39^. Microwave structures are typically sensitive to the permittivity of the dielectrics, which enables microwave sensing. Microstrip line is one of the most widely used transmission line structures in microwave systems, which constitutes of a signal line, a dielectric substrate and a ground plane. By embedding microfluidic channels into the substrate as shown in Fig. 7(b), a liquid-reconfigurable microstrip line can be easily designed. These microstrip lines can be modeled with the equivalent circuit in Fig. 7(a), in which the capacitance value is changing as a function of the permittivity values of the liquids filling the channel. As the characteristic impedance of the line depends on the capacitance of the tunable capacitor, its value varies with the permittivity of the fluids inside the microchannel. As a proof-of-concept, two inkjet-printed microstrip line prototypes of this topology were fabricated with the dimensions listed in Table 1. Figure 7(c) shows the photo of the fabricated microstrip line 2 that features only minor dimension differences in the height of the embedded microfluidic channel and the width of the signal line, with respect to line 1. Microstrip line 1 has been initially designed (empty channel) for a 50 Ohm characteristic impedance, which is the most common/reference impedance value in microwave designs. For the different filling liquids in Table 2 with a relative permittivity up to 5, the impedance can be tuned to as low as 31 Ohm (38% shift) as shown in Fig. 7(d). Similarly, microstrip line 2, which has an 87 Ohm default impedance, can be tuned down to as low as 67 Ohm (23% shift) with the liquids in Table 2, as clearly shown in Fig. 7(d). Due to its larger channel height and wider signal line width, Microstrip line 1 is more sensitive to different fluids inside the channel compared with line 2. This liquid-reconfigurable microstrip topology could be potentially utilized in numerous applications including reconfigurable impedance matching in communication systems as well as wireless biosensing and water quality monitoring.

Based on the above introduced liquid-reconfigurable impedance-tunable microstrip line topology, a step impedance low pass filter was built alternating high- and low-impedance transmission line sections. Figure 7(e,f) shows photos of a fabricated prototype and Fig. 7(i) lists the dimensions of the structure. This low pass filter would allow the propagation of low-frequency signals (under 13 GHz, passband), while attenuating signals at higher frequencies (stopband). The amount of attenuation strongly depends on the characteristic impedance of the transmission line sections, which varies for different fluids inside the channel. As a consequence, the fluid permittivity can be effectively sensed through the amount of the signal attenuation at higher frequencies while the signal attenuation at lower frequencies (passband) can be used as a calibration. The measured signal attenuation (or insertion loss, S21) (Fig. 7(g)), matches the electromagnetic simulation results and demonstrates an excellent sensitivity as high as 4 dB/*ε*_*r*_. The signal attenuations values of the sensor when filling the channel with hexanol and ethanol featuring a relative permittivity difference of only 2.2 can be easily distinguished by a larger than 2dB difference, thus verifying the potential applicability of this inkjet-printed sensor topology to various fluid sensing applications, such as laboratory analysis, wireless liquid quality monitoring and distributed healthcare.

## Discussion

In the past, microfluidics used to be an expensive tool due to their high cost fabrication process. Nevertheless, in order for microfluidics to be extensively applied in applications such as Lab-on-a-Chip and distributed healthcare, they need to be fabricated in short time at very low cost. The fully inkjet-printed microfluidics reported here provide a disruptive solution to cost-effective rapidly prototyped 3D microfluidics channels, thus enabling disposable sensors in distributed health care and chemical/bio analysis. This process is faster than soft-lithography, features a better resolution compared to normal 3D printers, and can enable fully 3D microfluidic topologies in virtually every material, such as paper. The fabrication process relies on a single platform, which is an easy-to-use industry-level inkjet-printer. This approach features an extremely low cost, less than $0.1 per device, and a short production period, only few hours for hundreds or thousands of prototypes. Due to the inherent additive nature of the proposed process, it can be directly implemented on virtually any substrate/device - even on top of another microfluidics chip - as we have successfully demonstrated for glass, copper, and different flexible polymers in this paper. During the evaluation of the process through the fabrication of various microfluidic prototypes, we characterized the process and demonstrated its capability to print a 5 *μ*m minimal/20 *μ*m optimal horizontal resolution and down to 4.6 *μ*m/layer in the vertical direction with less than 0.4 *μ*m fabrication variation, which is superior compared to other AM techniques such as 3D printing so far^10^,^11^,^12^,^13^. The minimum fabricated cross-section of the inkjet-printed microfluidic channel was a 60-*μ*m-width and 0.8-*μ*m-height channel (38 *μ*m^2^ cross-section area), which is smaller than one thousandth of the smallest 3D printed channel cross-section area^10^,^11^,^12^,^13^. The maximum printing area of the utilized inkjet printer was 210 mm × 315 mm, which could accommodate virtually any microfluidics devices and could enable a rapid and scalable fabrication by inkjet printing multiple microfluidics prototypes at the same time. Any channel with a cross-section aspect ratio larger than 10 can be easily implemented with the proposed direct printing approach. When printing microfluidics structures using SU-8, special attention has to be paid to the coffee ring effect as well as to providing a sufficient UV exposure, otherwise cracks may happen thus ruining the quality of the printed microfluidics. Single-material fully 3D microfluidics topologies can be easily fabricated using this inkjet-printing process with a level-by-level methodology similar to 3D printing, in which the fabrication of each individual level is independent of other levels. To further demonstrate the potential applications, we prototyped and evaluated several examples of typical microfluidics structures as well as sensors with integrated electronics. Silver nanoparticle ink was printed and sintered for high conductivity (e.g. 0.01 Ohm/square resistance) circuits. The prototyped microfluidics structures have featured a very good pressure handling capability and a long term endurance. An excellent flexibility and transparency further extend their applicability to wearable and optical domains. The proposed fully inkjet-printing process handles both microfluidics and electronics fabrication very well, which makes it a perfect candidate for the manufacturing of microfluidics sensors with integrated electronics. Overall, the proposed process can be used to fabricate fully inkjet-printed microfluidics for various applications including but not limited to wearable sensors, biomedical assays, chemistry analysis and micro-fabrications.

## Methods

### Inkjet-printing conditions

All the inkjet printing was conducted in a Dimatix DMP-2831 inkjet printer (Fujifilm Dimatix, Inc., Santa Clara, USA) with a 1.5-mL-capacity cartridge which had a 10 pl drop volume (DMC-11610, Fujifilm Dimatix, Inc.). A 20 *μ*m drop spacing and 40 °C platform temperature were chosen to balance the printing speed and performance.

### Inkjet-printing inks

Three different inks were involved in fabricating the fully inkjet-printing microfluidics or microfluidics-based sensors. The silver ink was SunTronic silver nanoparticle ink from Sun Chemical Corporation (Parsippany, USA), while PMMA ink and SU-8 ink were home-made. The PMMA ink was made by dissolving PMMA powder into a mixture of anisole and dimethyl sulfoxide (DMSO) with anisole dissolving the PMMA powder and DMSO optimizing the ink drop’s surface tension and viscosity. All these chemicals including PMMA, anisole and DMSO were from Sigma-Aldrich Corporation (St. Louis, USA). The SU-8 ink was a blend of SU-8 2002 and SU-8 2005 (Microchem Corporation, Westborough, USA). The ratio of two different viscosity SU-8 was carefully tuned to enable successful printing. All inks were pre-filtered through a 0.2 *μ*m syringe filter to prevent potential nozzle clog. Ultrasonic cleaning bath (Branson 1510 ultrasonic cleaner, Danbury, USA) was used in both blending the inks and etching the channels.

### Inks curing

To print solid structures out from liquid state inks, a curing step is very important for every inkjet-printing process and needs to be done right after the printing. The PMMA ink was dried at 120 °C on a hot plate for 40 minutes to ensure all the solvent was evaporated. The SU-8 ink was firstly soft baked at 95 °C on a hot plate to evaporate the solvent. Then it was cross-linked by a UV exposure and a post-exposure bake at 95 °C. Finally we hard bake the cross-linked SU-8 with a slow temperature raising to 150 °C to further enhance the mechanical strength of the printed structure and anneal any surface cracks during developing. The appropriate time and exposure needed in this curing process highly depends on the thickness of printed SU-8 and can be found at the manual provided by the manufacturer^38^. For a thicker SU-8 layer, over exposure of the SU-8 on top is required to fully cross-link the bottom SU-8, which could result in a brown color as in Fig. 5(b). The silver ink was sintering at 120 °C in a Thermo Scientific oven for 1 hour to achieve a good conductivity. The surface profile of the cured ink trace was measured by a contact profilometer (Alpha-Step D-100 Stylus Profilometer, KLA-Tencor, Milpitas, USA).

### Surface cleaning and treatment

Before depositing the ink, it is very important to ensure that the substrate surface is clean, in order to avoid roughness and surface energy varying due to impurity on the surface. Thus, we cleaned the substrate surfaces with Acetone and Ethanol fluids in sequence and drying with air fluids every time before printing. Every substrate has its own surface energy and so does inks. Good match of these two energies, which presents as an appropriate contact angle, is significant for a successful printing. Therefore, surface treatment to adjust the contact angles for the ink in certain substrates may be necessary. For example, before printing SU-8 on various substrates including cured SU-8, glass and LCP, the substrates were treated at UVO cleaner (Jelight company Inc, Irvine, USA) for 90 seconds.

### Substrates

Glass in Fig. 6(c) is from Corning (Corning, USA). LCP with copper substrates in Figs 6(d) and 7 were ULTRALAM 3850HT Laminates (Rogers, Rogers, USA) with its flexibility and excellent high frequency properties. Due to its high transparency and flexibility, PET film (Melinex ST506/700, Teijin DuPont films, Tokyo, Japan), was used in Fig. 6(a,e). Another flexible polymer, Kapton polyimide film in Fig. 6(b), was from DuPont (Circleville, USA).

### Electronics design, characterization and measurement

The electronic sensors in this paper (in Fig. 7) are operating in microwave frequencies so microwave simulators, and measurement devices are involved. Agilent Advanced Design System (ADS), a Radio Frequency (RF) circuits simulator, was used in circuit simulation and optimization. Ansoft High Frequency Structural Simulator (HFSS), a finite element electromagnetic (EM) simulator, was utilized in full-wave EM simulating and further tuning the design. Due to their small sizes, the sensors were measured by a probe station (Cascade, Beaverton, USA) as shown in Fig. 7(h). The frequency domain responses of the sensors were read by an Anritsu 37369A Vector Network Analyzer (VNA) (Kanagawa, Japan).

### SEM analysis

The cross-sections of the microfluidics were created by simple cutting, aided with a ruler, at appropriate locations through the cured Su-8 polymer and the LCP substrate with a razor blade. Scanning electron microscopy was conducted with a field emission scanning electron microscope (Leo 1530 FEG SEM, Carl Zeiss SMT Ltd., Cambridge, UK).

## Additional Information

**How to cite this article**: Su, W. *et al*. Fully inkjet-printed microfluidics: a solution to low-cost rapid three-dimensional microfluidics fabrication with numerous electrical and sensing applications. *Sci. Rep*. **6**, 35111; doi: 10.1038/srep35111 (2016).

## Figures and Tables

**Figure 1:**
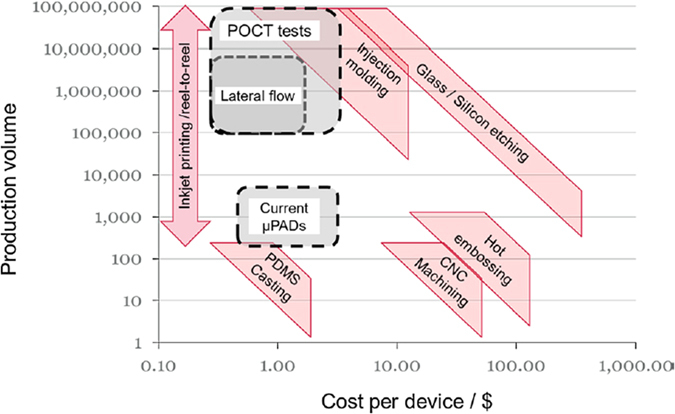
Cost and volume comparison for common Lab-on-A-Chip fabrication technologies. Reproduced from^17^ with permission.

**Table 1:** Dimensions of the two embedded-microfluidics liquid-reconfigurable microstrip line prototypes.

Name	Channel height (*ch*)	Channel width (*cw*)	Substrate height		Signal line width
			bottom (h1)	top (h2)	
microstrip line 1	50 *μ*m	600 *μ*m	11 *μ*m	66 *μ*m	0.32 mm
microstrip line 2	30 *μ*m	600 *μ*m	11 *μ*m	66 *μ*m	0.12 mm

**Table 2:** Relative permittivity of air and various channel-filling liquids at 10 GHz at room temperature^39^.

Liquid	Relative permittivity
Air	1
Hexanol	2.8
Ethanol	5
